# Experiences of health service access: A qualitative interview study of people living with Parkinson's disease in Ireland

**DOI:** 10.1111/hex.13901

**Published:** 2023-11-05

**Authors:** Emma O' Shea, Aphie Rukundo, Geraldine Foley, Tony Wilkinson, Suzanne Timmons

**Affiliations:** ^1^ Centre for Gerontology and Rehabilitation, School of Medicine University College Cork Cork Ireland; ^2^ Discipline of Occupational Therapy, School of Medicine Trinity College Dublin Dublin Ireland; ^3^ Cork Parkinson's Association Parkinson's Association of Ireland Dublin Ireland

**Keywords:** inequitable access, multidisciplinary approach, Parkinson's disease, qualitative, telemedicine

## Abstract

**Background:**

People with Parkinson's disease (PD) do not always access specialist outpatient services in a timely manner in Ireland. The perspectives of people living with PD, relating to service access, are largely absent in the existing literature.

**Aim:**

To explore experiences of PD service access for people living with PD, using a qualitative approach.

**Methods:**

Purposive maximum variation sampling was used. Semi‐structured telephone interviews were conducted with 25 service users, including people with PD (*n* = 22) and supporting carers (*n* = 3). Informed consent was obtained from all participants. Interviews ranged in duration from 30 to 90 min. Data were managed in NVivo 12 and interpreted inductively using thematic analysis. The researchers were reflexive throughout the research process. The Consolidated Criteria for Reporting Qualitative Research checklist was employed to maximise transparency.

**Results:**

The findings highlight several key barriers to and facilitators of equitable and timely service access. Three key themes were identified comprising experiences of PD service access including ‘geographical inequity’, ‘discriminatory practices’, and ‘public and private system deficits’. Together, these themes illustrate how a two‐tiered and under‐resourced health system lacks capacity, in terms of infrastructure and workforce, to meet PD needs for both public and private patients in Ireland.

**Conclusions:**

These findings point to problems for PD care, relating to (i) how the health system is structured, (ii) the under‐provision and under‐resourcing of specialist outpatient PD services, including medical, nursing, and multidisciplinary posts, and (iii) insufficient PD awareness education and training across health settings. The findings also show that telemedicine can provide opportunities for making access to certain aspects of PD care more flexible and equitable, but the feasibility and acceptability of technology‐enabled care must be assessed on an individual basis. Implications for policy, practice and research are discussed.

**Patient or Public Contribution:**

The design and conduct of this study were supported by an expert advisory group (EAG) of 10 co‐researchers living with PD. The EAG reviewed the interview schedule and the protocol for this study and provided detailed feedback from their perspective, to improve the methods, including the interview approach. The group also reviewed the findings of the study and contributed their insights on the meaning of the findings, which fed into this paper.

## 1 INTRODUCTION

Parkinson's disease (PD) is a multisystem neurodegenerative condition, characterised by bradykinesia, rigidity, resting tremor, and postural instability, along with a wide range of nonmotor symptoms, including but not limited to neuropsychiatric symptoms, sleep disturbance, autonomic dysfunction, and fatigue.^1^ Current global PD prevalence estimates show that there are six million people living with the condition, which is 2.4 times larger than the global prevalence reported in 1990.^2^ Importantly, age‐standardised PD prevalence rates increased by approximately 22% over this same period. Future projections indicate that prevalence will rise to 9.3 million by 2030.^3^ According to Dorsey et al.,^2^ the continued growth in prevalence can be attributed to population ageing, improved detection/diagnostic processes, as well as increased exposure to some risk factors (e.g., pesticides, certain metals). The estimated total cost of PD care at the European level is €13.9bn annually,^4^ which the authors consider a ‘conservative’ evaluation. The high cost burden is consistent with findings from studies from the United States, Canada, and the United Kingdom, showing that compared to people without PD, those with PD use significantly more healthcare, i.e., more emergency department, in‐patient, rehabilitation and care home admissions, and lengthier hospital stays.^5^, ^6^, ^7^

In Ireland, as in many Western, high‐income countries, national policy aims to provide timely access to integrated and interdisciplinary PD care.^4^ According to a report by the Neurological Alliance of Ireland,^8^ detailing the findings of a national survey of neurology centre resourcing, the following problems exist: excessive waiting times for diagnostics; lengthy neuroimaging waiting lists; significant neurology and multidisciplinary team staffing deficits; and a lack of neurorehabilitation services, including for PD. Such findings regarding PD care capacity are not unique to the Irish system. The above findings reflect deficits also reported in other countries, which act as significant barriers to providing integrated PD care.^9^, ^10^, ^11^ Given the projected growth in PD prevalence, we urgently need to focus on capacity‐building in services to create timely and equitable health service access for an expanding PD population.

While we have some understanding of deficits in PD service provision, we have a much poorer picture of the patient experience, and their perspectives on service access. A multicountry survey of 1775 people with PD across 11 European countries reported that patient satisfaction was especially low (48%) regarding the availability and accessibility of treatment when needed.^12^ Similarly, a 2014 patient survey (*N* = 601) in the Irish context found that satisfaction with neurology‐led care was low across the following indices: information made available about the condition (51%); support for linking in with other services (42%); getting referrals to other services (39%); and the provision of psychological support (32% satisfied).^13^ While these insights are valuable and relate to the Irish context, they are not specific to PD care.

To our knowledge, there has been just one study by Fox et al.^14^ that has explored PD patients' needs and healthcare experiences in Ireland, from a palliative care perspective. Semi‐structured interviews were conducted with PD patients (*n* = 19) and family carers (*n* = 12). Amongst a range of other key issues, people with PD often face prolonged diagnostic processes. Postdiagnosis, PD patients were often unsure of what services or support were available to them or how to gain access to services. The authors concluded that this, along with other findings, reflect a fragmented, and primarily biomedical model of PD care, which alone cannot cater to the complex needs of people living with PD.

The importance of conducting research with patients has become more evident in recent years, as there is greater recognition of the natural expertise patients possess relating to their personal experiences with their condition. However, the existing research does not provide rich information about the experience of accessing specialist PD services from the patient perspective. To address this gap, we aimed to explore experiences of PD service access for people living with PD across Ireland.

## 2 METHODS

Semi‐structured qualitative interviews were conducted with people with PD in the Republic of Ireland between April 2020 and January 2021. This study is presented according to the Consolidated Criteria for Reporting Qualitative Research.^15^ It is part of a wider programme of health services research which aims to map PD service provision in Ireland, assess service performance against quality indicators of PD care, and examine the experiences of patients as regards accessing and using PD services. As per the preferences and advice of the expert advisory group (co‐researchers), this research was designed to be accessible, tangible and impact focused. We adopted a pragmatic approach to the overall programme of research, whereby experiences and perspectives of people living with PD, and accessing/using PD services, could be synthesised and translated usefully for end knowledge users (e.g., department of health, and health service decision‐makers and planners).

### 2.1 Recruitment/sampling

Purposive maximum variation sampling was employed to ensure diversity among the sample (e.g., sex, rural/urban, age, age at diagnosis, years since diagnosis, etc.). While people with PD were our focus, three family carers were also included, to support people with advanced PD to share their experiences. Inclusion criteria were having a formal diagnosis of PD (or supporting a person with PD to participate) and being (i) currently resident and (ii) availing of healthcare in the Republic of Ireland. A phone call was arranged with prospective participants who met the basic inclusion criteria to determine if the interested person fit the sampling frame. As data collection progressed, the sampling frame criteria became more specific. We specifically searched for people with underrepresented demographic characteristics within our working sample (e.g., those living in rural areas across each province; those with younger‐onset PD; those diagnosed >10 years ago; those not linked in with support organisations/groups; people from minority groups) and began excluding those who were overly similar to our existing sample.

Recruitment was multipronged and achieved through multiple routes including voluntary support groups (Parkinson's Association of Ireland, Move4Parkinson's, Young Parkinson's Ireland), social media outlets (Facebook, Twitter, Instagram), local newspaper, magazine and radio advertising, and posters in nursing homes and GP offices. A particular emphasis was placed on social media to reach this cohort, given that most of the target population was ‘cocooning’ due to the COVID‐19 global pandemic. In line with our sampling strategy, we also advertised with over 20 organisations representing/supporting minority groups (e.g., Irish Travellers, people who are homeless or in direct provision, and LGBT+ communities).

### 2.2 Data collection

To accommodate the COVID‐19 public health restrictions, all interviews were conducted by telephone. Information sheets, consent forms and data protection notices were provided to potential participants and were returned electronically or via post. Before conducting interviews, participants were again asked for verbal consent to record the interview for the purpose of transcription. Participants were reminded of their right to stop the interview at any point, whether for a break, medication consumption, or to withdraw participation. Interviews ranged from 30 to 90 min. Where interviews exceeded 1 h, and the participant wanted to continue, a brief recess was taken.

An interview schedule was developed in partnership with the research team (which included people living with PD). This was used as a guide to benchmark key areas of the research. Topics included: diagnostic pathway; delivery of the diagnosis; information sharing; service availability; allied healthcare professional access; and care needs and preferences. This schedule was used flexibly to allow participants to share salient aspects of their experiences with health services even if these experiences were not initially focused on in the interview schedule. A copy of the interview schedule was provided to participants in advance. All interviews were recorded using a digital audio‐recorder.

### 2.3 Data management and analysis

Interviews were immediately transcribed, and the audio files deleted thereafter. Anonymised codes were given to the audio and written data, to uphold confidentiality. These were stored on an encrypted, password‐protected hard drive and on the cloud (secure University‐hosted server with daily back‐up). Any personal information that could potentially render participants identifiable was redacted. Interviews were transcribed verbatim. Data were managed using NVivo 12.

Braun and Clarke's^16^ inductive thematic analysis was employed to analyse the data. This entailed the rereading of transcripts and making memos before beginning the coding process. Initially, the first 40% (10/25) of interviews were coded using a bottom‐up approach. Meaningful sections of data were open‐coded in relation to the research question. The initial codes were used to inform coding of later transcripts (i.e., a coding frame) and then codes were iteratively updated as more data were analysed. ‘Updated’, in this context, comprised the removal/merging of duplicate codes, renaming and restructuring of initial codes, and the addition of novel codes, with each additional interview. This process continued until all data were coded.

Subsequently, the codes and the data comprising them were compared within and across interviews. This process informed the grouping of codes into categories, which over multiple iterations, were crafted into defined themes. We were intentional about engaging with the data in an interpretive manner. This was achieved through team discussions about identified patterns within the data, and their meaning and implications for the research question. Team discussions also focused on the issue of reflexivity. Researchers kept a journal of their perspectives throughout data collection and data analysis, which were discussed with senior members of the team. This helped challenge potential biases and assumptions held by the analysts.

## 3 RESULTS

In total, 25 participants were included in this study. The median participant age was 64 years (interquartile range = 17.5 years), with a minimum of 31 and maximum of 90 years. The median number of years since diagnosis was 5 (interquartile range: 7.25 years; minimum = 1 year, maximum = 20 years). Most interviewees were married/had a partner (68%), 16% were divorced/separated, 8% were widowed, and 8% were single. Regarding occupation, 52% were retired, 8% semiretired, 28% were employed, 8% self‐employed, and 1 (4%) person reported being a ‘homemaker’. Other sociodemographic characteristics of the sample are outlined in Table 1.

**Table 1:** Interviewee demographic characteristics.

Participant		Sex	Urban/rural	Public/private patient
1	PD	Female	Urban	Public
2	PD	Female	Urban	Private
3	PD	Male	Rural	Public
4	PD	Male	Urban	Private
5	PD	Female	Urban	Public
6	PD	Female	Rural	Private
7	PD	Male	Rural	Public
8	PD	Female	Urban	Public
9	PD	Female	Urban	Public
10	PD	Male	Urban	Public
11	PD	Female	Rural	Public
12	PD	Female	Urban	Public
13	PD	Female	Rural	Public
14	PD	Female	Rural	Public
15	PD	Male	Urban	Public
16	PD	Female	Rural	Public
17	PD	Male	Urban	Public
18	PD	Female	Rural	Private
19	PD	Female	Rural	Private
20	Carer	Female	Rural	Private
21	PD	Male	Rural	Private
22	Carer	Female	Rural	Private
23	PD	Male	Rural	Private
24	Carer	Female	Urban	Private
25	Carer	Male	Rural	Public

Abbreviation: PD, Parkinson's disease.

Three key themes were identified as comprising the experiences of people living with PD, in relation to PD service access: ‘geographical inequity’, ‘discriminatory practices’ and ‘public and private system deficits’. The final theme regarding health system deficits is comprised of two subthemes, that is, ‘inadequate public diagnostic services’ and ‘poor multidisciplinary team access for private patients’. The themes and subthemes are presented below.

### 3.1 Geographical inequity

A significant factor influencing patterns of access to PD services related to the distribution of outpatient services across the country. For people with PD, especially those living rurally, the concept of a ‘postcode lottery’ was recurring. A substantial variation in service availability was evident across urban and rural areas, with the latter being exceptionally under‐served.

*We are a bit disadvantaged … We don't get as much from services as those who live in big towns or cities … There isn't much of an effort to cater to people like me…*

*It would be good if I didn't have to travel so far…*

The lengthy travel period to attend some services acted as a significant barrier for people with PD who often require multidisciplinary team input to adequately manage their symptoms.

*I did see a physio. It was in the clinic, which was kind of disappointing because we live an hour and a half, two hours away … We don't mind coming to the city for doctors' appointments, but it really didn't work going to a physio that far away … It's a shame really because I haven't found anything closer to home*.

The considerable regional variation in PD service provision has left some rural communities with no choice but to self‐organise and pay out‐of‐pocket for PD/neurology nurse specialists and/or clinical therapists to travel and run local information sessions, clinics and/or classes.

*We're missing out on all that … Once we had a lady come to us out of the city and she came here to the hall and it was for movement and balance*.

In contrast, two interviewees living rurally, near smaller, regional public hospitals experienced shorter waiting times between appointments, timely facilitation of unplanned reviews and greater continuity of care than can be achieved in some tertiary urban hospitals covering larger catchment areas.

*I get a great deal more time with him [*consultant*], and it's the same person always because he is the only one …. He knows me*.

Telehealth was suggested as a potential method for offsetting some of the geographical inequity in terms of accessing specialist PD care, both in terms of medical management and multidisciplinary team care. Several people with PD in this study were hopeful that the adaptations made in response to the COVID‐19 pandemic would remain sustainable options going forward.

*There is probably nothing like a disaster or pandemic to force the health service to get creative … I really hope that they don't forget about us on the outskirts though … It would be a great thing in my opinion if we could stay connected more to the professionals, using technology*.

Conversely, others were concerned that telehealth might replace face‐to‐face interactions, especially for those living greater distances from specialist PD services. This prospect was not acceptable, with some pointing out the limitations of videoconferencing technology when it comes to assessing motor symptoms and when discussing sensitive personal issues.

*It wouldn't be the same quality of assessment, because it is a movement disorder*

*I don't want that to be the way everything goes … For instance I don't want to do a counselling session on zoom*.

### 3.2 Discriminatory practices

Discrimination was a common healthcare experience for many with PD, at two distinct but sometimes intersecting levels; first, based on age, and second based on perceived severity of disability.

Regarding age, those with young‐onset PD noted how their pathway to diagnosis was sometimes complicated by the knowledge and beliefs of general practitioners (GPs), particularly those that characterise PD as a disease of older age. Several young‐onset PD interviewees disclosed that their symptoms were initially dismissed (e.g., as ‘nerves’) or simply not recognised as PD‐related because GPs did not consider a differential diagnosis of PD to be consistent with the patient's age.

*[The GP said] ‘you're lacking in nutrients and vitamins’. So he put me on [supplement] and he left me off … Then I was admitted into hospital … I kept collapsing, having blackouts. And I was severely fatigued. I lost weight and the doctors were just like, ‘Tell us what you're taking?’… As in, they meant [sic: illegal] drugs*.

*I went to the GP first—that was not a great experience. She seemed to think my age meant it was unlikely that it was anything other than anxiety*.

Postdiagnosis, discrimination experiences are also common. Age appears to be considered a coarse barometer for need, in terms of accessing clinical therapies, for both motor and nonmotor symptoms. Indeed, some people with young‐onset PD seeking referrals from health and social care professionals were told such referrals were not yet appropriate.

*I haven't seen a speech and language therapist … [Consultant] said ‘It's only for people in their 50's*+ …*’ What's the point of waiting until I'm 50 when I'm already having problems?*

The second layer of discrimination affects people with PD of all ages. Many interviewees in this study were keen to take a proactive approach to their own care, in line with the principles of self‐management, but this is sometimes met with pushback, owing to the patients not being ‘bad enough’ yet to avail of services.

*I hear them saying, ‘You don't need physiotherapy’, for example, ‘because you're not* “*that bad*” *yet’*.

Even when referrals are made to clinical therapies, some participants reported that therapists distinguish between those who they perceive sufficiently disabled to warrant the need of services and those not disabled *enough* to warrant intervention.

*The service was really disappointing … I was seen by a basic grade physio who didn't have experience in Parkinson's … [They] said, ‘You're very high functioning so, you know, we don't need to see you.’ I was like, ‘Well, the whole point is getting it early?’. But that is not how Parkinson's services are set up, so I was discharged*.

While GPs and PD specialists of all disciplines often ‘prescribe’ physical exercise as one of the most effective facets of self‐management, several public patients highlighted that those who can afford gym or pool memberships and exercise classes are at a distinct advantage, over those who cannot.

*I love to swim, but again that is something that works out expensive … If there was some way of doing something like that … Like if it were even partly paid for, because I'd be scrambling trying to get the money together … If there was some sort of scheme, people could help themselves that way*.

Similarly, several private patients, some of whom admitted they could ‘just about’ afford health insurance, indicated a need for financial support for exercise programmes from their insurance companies.

*One thing that should be explored is what the private health insurance companies will do for Parkinson's patients to keep them active. Because I'm sure it makes a big difference to them whether we stay active or not—like with income protection insurance, there is a benefit for them, if they can keep you in work*.

### 3.3 Public and private system deficits

Many interviewees spoke to their own, and their peers' experiences, of the limitations and deficits of both the public and private health systems for PD care. The first subtheme below outlines the absence of sufficient capacity to enable timely diagnosis within the public health system, compared to the relatively swift pathway to diagnosis for private paying patients. The second subtheme illustrates that while being a private patient may have clear benefits in the diagnostic and early stages, private patients face substantial disadvantages as the disease progresses, owing to poor multidisciplinary team access.

Many private patients switch to the public system as their condition progresses, to gain access to the range of clinical therapies. Indeed, eight out of 23 interviewees with PD reported having switched from private to public care as their condition progressed, with one further participant indicating they were currently in the process of switching to the public system.

#### 3.3.1 Inadequate public diagnostic services

One of the most expressed concerns by people with PD related to the prolonged waiting periods to see a PD specialist, in the public system. The lengthy public waiting list is an active deterrent, leading many to use the private system for diagnosis. Indeed, many reflect on their choice to ‘go private’ as one of compulsion, rather than choice.

*It was fairly quick. I would say it was a month or something … It was private. It wasn't public … I would be forever going through the public system*.

*If my GP had referred me through the public system, I probably still wouldn't have a diagnosis*.

However, some interviewees could not afford to pay out‐of‐pocket, creating a distinct inequity for those with fewer financial resources, with some waiting 18–24 months, and one person with young‐onset PD waiting 4 years to obtain a diagnosis through the public system:

*I thought about going private, but you know we weren't in a position financially*.

The protracted waiting period was experienced as ‘limbo’ by some who suspected they were facing a neurodegenerative diagnosis, which can be emotionally taxing on the person and those close to them.

*I did go through hell, I couldn't … I spent most of my time crying for a couple of months … and my family … I don't know how they put up with me*.

A barrier to timely diagnosis faced by both public and private patients was the additional waiting time for medical imaging (e.g., magnetic resonance imagings, and specialist molecular imaging such as dopamine transporter scans), and/or the wait for a review appointment for the results of same. Several interviewees noted having to chase the imaging results themselves, indicating a point of significant fragmentation in the health service.

*I contacted his secretary … I found her extremely difficult … I was just ringing to know when the appointment was going to be arranged. So eventually I contacted the hospital myself, and there was nothing happening. So, I asked if they could they follow it up on their side. Eventually I got the DaT scan, but it was the way it was handled was horrendous. And being left second‐guessing for months*.

Additionally, it was evident that postdiagnosis, the wait times between outpatient follow‐up visits is significantly greater for those in the public system. One interviewee, who has sought help through both systems, summarised his experience:

*In the private system, I had a visit every six months, at least. Now I'm in the public system it has gone up to a year. And at the moment [due to the COVID‐19 pandemic], it's looking like it will be 18 months between visits*.

#### 3.3.2 Poor multidisciplinary team access for private patients

A common trend disclosed by interviewees was how some of the systemic inequity associated with being a public patient during the diagnostic process reverses somewhat postdiagnosis. While public patients reported longer wait times for diagnosis and subsequent follow‐up visits than private patients, they also reported superior access to the range of clinical therapies as their condition progresses.

*You're much better off being in the public system once you have a chronic disease, in terms of getting access to therapies*.

*I was diagnosed in a private hospital … I later transferred to the public system … Because the nature of Parkinson's is that you will need a lot of support. And the private health services aren't set up for that*.

While some acquired this insight through their own research and peer discussion, others noted that their nurse, consultant and/or GP, advised that a switch from private to public care was in their best interest, post‐diagnosis, for optimal symptom management.

*[Consultant] recommended that I get deep brain stimulation, but they needed me to come as a public patient. We're in the process of changing me to a public patient now … Otherwise, I'd miss out on occupational therapy, physiotherapy, language therapy … all of that*.

However, this insight is not always shared with patients diagnosed in the private system; thus, deepening inequalities in access to clinical therapies for long‐term private patients.

*[sic: Private Consultant] would often say things like, ‘Oh, you need to go to physiotherapy, or you need to talk to so and so …’ But he doesn't give you any names. So, I'm kind of left going ‘well, who do I go to?’*

Several interviewees in the public system who had been able to access multidisciplinary team input, indicated that this was due to their clinic having a dedicated PD nurse specialist that provides information and advice, and who can ordinarily function as a key gatekeeper and/or coordinator for multidisciplinary team referrals:

*The major difference since going public is that there was a nurse who we got to speak with and she gave us great information about different services … We didn't get that in the first place … She gave us her number and for the first time I felt like I had someone that I could potentially call if I had a problem or a question*.

While PD nurses are seen by many as being at the fulcrum of any holistic, interdisciplinary PD care model, many participants (both public and private) do not have access to a specialist PD nurse in the clinic that they attend.

*It's completely under‐resourced, but the resources that are available are wonderful and so professional. Say PD nurse specialists—we have nowhere near enough of them, and it's considerably less than what they have available in other countries*.

## 4 DISCUSSION

This qualitative study explored the experiences of PD service access for people living with PD across Ireland. Three key themes were identified as comprising the experiences of people living with PD, in relation to PD service access, that is, ‘geographical inequity’, ‘discriminatory practices’, and ‘public and private system deficits’. These findings highlight significant problems for PD care, relating to (i) how the health system is structured, (ii) the under‐provision and under‐resourcing of specialist outpatient PD services and multidisciplinary posts, and (iii) insufficient PD education and training across health settings. It is worth noting that although the challenges encountered by participants may have been somewhat exacerbated by the COVID‐19 pandemic, they have also long‐preceded the COVID‐19 pandemic, reflecting the ongoing capacity and resourcing issues identified in this study.

The Irish health system is commonly characterised as ‘two‐tiered’ in structure, such that those who can afford to pay privately, and/or have voluntary health insurance, typically gain speedier access to diagnosis and treatment than those in the public system.^17^ Ireland is an outlier compared to other European countries, where the universal coverage model is prevalent, in line with the recommendations of the World Health Organization,^18^ i.e., that everyone should be able to access the health services that they need, when needed, and at an affordable cost. While the 2017 ‘Sláintecare’ health policy outlined a 10‐year structural health service reform plan for Ireland, towards universal healthcare^19^ and had cross‐party political support, the commitment to full and timely implementation is still unclear. Parker et al.^20^ highlight how the COVID‐19 pandemic led to system‐wide, rapid pivots in care organisation and provision, and that we should use the key learnings from this to inform the shift towards universal healthcare.

In terms of the pitfalls of the two‐tier system, inequity is oft‐cited, particularly for those who cannot afford to pay for private care.^17^ However, this study demonstrates that the situation is even more nuanced in the context of PD care. As expected, most participants agreed that diagnosis is far less protracted in the private system, noting that the delays to first assessment in the public service are longer for people referred to a neurology‐led versus a geriatrician‐led PD service in Ireland. However, despite many opting for a speedier private diagnosis, a clear pattern of transfer from the private to the public system occurred postdiagnosis for several participants and was acknowledged as a well‐known phenomenon by others. While cost was a significant factor in this switch, access to integrated multidisciplinary teams was often the primary driver of decisions to switch from private to public care. As their condition progressed and a more holistic approach was required, some participants struggled to access the required range of health and social care professionals, and even device‐assisted therapies (e.g., deep brain stimulation). Thus, while using the private system bypasses some initial access issues, the utility and value of the private stream is called into question, in the context of advancing PD.

Related to this issue is the consistent under‐resourcing of public PD outpatient services, which has clear consequences for service access. Participants frequently noted the lack of PD specialists across disciplines, with the skills and expertise to appropriately manage the range of complex health issues that they faced. Those living in rural areas were often disadvantaged in terms of service availability locally, and many travelled great distances, incurring significant out‐of‐pocket travel, subsistence, and accommodation costs to see PD specialists, in both the public and private systems. This issue also has some layers of complexity, as two participants from unconnected rural areas, both in the public system, indicated they had frequent routine access and rapid unplanned access to their PD consultant in their local (regional) hospital. This may reflect medical consultants in some rural hospitals having a special interest in PD care, and it does lend weight to the ability of services outside of large tertiary centres being able to provide a flexible service locally for people with PD.

A report from the Neurological Alliance of Ireland^8^ outlined concerns regarding critical understaffing across all neurology services in Ireland in medical, nursing and clinical therapy disciplines, and the systems' poor capacity in terms of overall infrastructure, access to brain imaging, and support for everyday living. The authors report limited additional consultant neurologist appointments between 2015 and 2020; clinical nurse specialist staffing was at 44% of the required national level; and multidisciplinary teams were either understaffed or unavailable across all neurology centres. More specific to PD, the survey reported that there were just six PD‐specific nurse specialists, compared to the estimated minimum population need (i.e., *N* = 30) as per the National Institute for Health & Care Excellence PD guideline's^21^ recommendation that all people with PD should have access to a PD nurse specialist.

Moreover, the NAI report spotlighted that neurology services are ‘critically reliant on a network of not‐for‐profit patient organisations' which provide services and supports in the community. This was reflected in the experiences of participants in this study, many of whom noted that the only support they receive in the community is organised and provided through PD patient organisations (e.g., The Parkinson's Association of Ireland, Move4Parkinson's). These organisations are also dependent on precarious government funding.

While the evidence across specific interventions is nuanced, multidisciplinary interventions can be effective for a wide range of PD outcomes.^10^, ^22^, ^23^ However, maintaining a comprehensive and dedicated multidisciplinary team in a single outpatient PD service can be expensive, and some experts question the feasibility and sustainability of this model.^9^ Several authors have proposed that this ‘expert centre’ model could be supplemented with community‐based integrated PD care networks, arguing that this is a more person‐centred approach to symptom management, which minimises patient travel and cost burden.^9^

In the Irish system, building capacity for specialist PD care to be delivered in local communities may be one way to reduce the current inequity associated with travel to larger, tertiary hospitals for outpatient care. Satellite clinics, either consultant‐ or nurse‐led, may provide an opportunity to begin such a shift to community‐based care. Evidence from Australia shows that providing PD nurses to work in regional communities confers significant cost benefits, with hospital cost reductions of up to $8600/person over a 3‐year period.^24^ In addition, creating regional posts for allied health professionals with specialist PD training is necessary. This is important, given the view from participants in the current study that non‐specialists do not confer as much benefit, and arguments that PD‐specialised clinical therapists will achieve better patient outcomes.^25^, ^26^, ^27^, ^28^ The Proactive and Integrated Management and Empowerment (PRIME) Parkinson's model of care, has been specifically designed to manage PD issues proactively, to deliver integrated multidisciplinary care, as well as to empower patients to engage in greater self‐management.^25^, ^29^ A clinical trial evaluating this new model is presently underway in the United Kingdom to evaluate a wide range of patient and carer outcomes, and cost‐effectiveness.^30^

Such a modified approach could be further supplemented using technology, as appropriate, to facilitate PD service access and to obtain objective measurements of motor fluctuations.^31^, ^32^ Recent research shows that PD patients are largely positive about wearable devices and their perceived benefits in symptom monitoring and management, for example, motor dexterity.^33^ Such ‘technology‐enabled care’ can include, for example, remote consultations and exercise interventions, and is increasingly incorporating the use of mobile health applications and wearable sensor devices for self‐management purposes as more validated tools come on stream.^9^, ^10^, ^23^, ^34^ The disruption caused by the COVID‐19 pandemic presented a marked opportunity to accelerate changes in PD care provision.^34^ In addition, there is potential for substantial developments in PD diagnostics and care, using machine‐ and deep‐learning, and artificial intelligence‐enabled technology.^32^, ^35^ Further research is needed to understand which aspects of care are most amenable to telehealth and other technological advancements. Cybersecurity and the wider regulatory landscape also remain central considerations.^31^ Finally, given the concerns and preferences of some patients that technology should not entirely replace in‐person care interactions, it is important that the acceptability and feasibility of such technology is individually assessed for each patient.

Regarding the discriminatory clinical practices outlined in this study, the provision of PD awareness education for health and social care professionals is paramount. The National Clinical Programme for Neurology in Ireland acknowledges that greater literacy and skill around PD recognition, management and specialist referral is needed amongst generalists within the health system. However, to tackle some of the prominent biases relating to the age of onset and facilitate timely access to allied health professionals for specialist PD care, more PD education is required across the disciplines and healthcare settings that are most likely to see a patient with PD. Of note, the ParkinsonNet initiative has previously developed PD‐specific clinical guidelines for physiotherapy,^36^ occupational therapy,^37^ and speech and language therapy services.^38^ According to the National Clinical Programme for Neurology report (2019), there has been some uptake of training in the physiotherapy guidelines, which is welcomed progress.

### 4.1 Limitations

This study included a sizeable purposeful sample and data saturation was reached. However, some limitations are noted. Data collection for this study was initially planned to be in‐person. However, the pandemic and the associated restrictions forced us to conduct interviews remotely. While we obtained rich data, it is possible that some meaning was lost through the remote channels of communication. As noted, we aimed for a maximally diverse sample, however, it proved exceptionally difficult during the COVID‐19 pandemic to recruit harder‐to‐reach groups (e.g., homeless, LGBTQ+, Irish Travellers, ethnic minorities, etc.) and so minority groups are not represented in this data. In addition, only English‐speaking people with an established PD diagnosis were included. Therefore, the findings of this study likely do not reflect the full range of experiences relating to PD care access.

## 5 CONCLUSIONS

This study has explored the experiences of PD service access from the patient's perspective. The findings highlight several barriers to and facilitators for equitable and timely service access, along with associated policy and service planning recommendations. The access‐related experiences of people with PD make a strong case for the need to implement universal healthcare in Ireland because neither the public nor the private health systems facilitate optimal PD care access. Substantial additional resources are urgently needed to create the infrastructure and the workforce needed to accommodate the growing population‐level need for PD care and to offset the reliance on the voluntary sector. Better PD awareness is also needed amongst generalist healthcare professionals across health settings to minimise bias relating to age and the severity of disability. Telehealth has proven a useful and acceptable means of communication throughout the COVID‐19 pandemic for health and social care needs. Further research is needed to understand which aspects of PD care are amenable to telehealth and other technological advancements.

## AUTHOR CONTRIBUTIONS

Emma O' Shea and Aphie Rukundo collected the data. Tony Wilkinson supported recruitment. Emma O' Shea and Aphie Rukundo analysed and interpreted the data. Emma O' Shea and Suzanne Timmons drafted the manuscript, with support from Geraldine Foley. All authors reviewed and gave feedback on the manuscript.

## CONFLICT OF INTEREST STATEMENT

The authors declare no conflict of interest.

## ETHICS STATEMENT

Ethical approval was obtained from the local research ethics committee (ECM 4 (m) 10/03/2020).

## Supporting information

Supporting information.

## Data Availability

The data that support the findings of this study are available on request from the corresponding author. The data are not publicly available due to privacy or ethical restrictions.
