# Supplementary material for: Experiences of health service access: A qualitative interview study of people living with Parkinson's disease in Ireland
Source: Health Expect. 2023 Nov 5;27(1):e13901. doi: 10.1111/hex.13901 (PMC10726277; doi:10.1111/hex.13901)
Supplement: Supplementary file 1 — Supporting information. [file HEX-27-e13901-s001.docx]

**Interview Topic Guide**

**Introductory questions:**

- Can you tell me a bit about yourself and your Parkinson’s?
- How long have you had PD for?
- How are you doing at the moment?

**General:**

- Can you please tell me about the PD service you attend?
- What do you think of this service?
- What’s good about it?
- What’s not so good about it?
- When you attend, who do you see at each visit? (e.g., consultant, physiotherapist, etc.)
- Overall, what is your level of satisfaction with the service?

**Diagnostic visit:**

Thinking back to when you/the person was first diagnosed *(omit if diagnosis was not made in Ireland):*

- How was that process for you?
- How did you feel after being told your diagnosis?
- What did you need most at that time?
- Did you feel you got what you needed from the service at that time?
- What was done well on that first visit?
- What could have been improved?
- How satisfied were you with the level of information that you got at the time? Did it give you what you needed? (Please explain)
- Did you get written information?
- Were you told about support groups?

**Unscheduled reviews:**

- Have you ever run into trouble or had issues with your PD in between scheduled visits at the clinic?
- If ‘yes’ - Was the clinic able to offer you any support?
- If ‘no’ – What do you think you would do in that situation?
- Have you any other suggestions for how the clinic could help in this event?

**Last visit to PD clinic:**

 Thinking back to the last visit….

- How long ago was your last visit to the PD clinic?
- What did you expect to happen at that visit?
- What did you hope to get from the visit?
- What was done well? What could have been improved?
- How do you travel to the clinic? Is travelling/parking an issue for you?
- Was getting into the clinic (building) an issue for you?
- How long did you have to wait to be seen beyond your appointment time?
- How were you treated by staff there? How was the communication with you?
- Is there a special nurse at the clinic? How do you find that (if present)?
- How do you find your doctor/consultant? Can you tell me more about XXX?

**Multidisciplinary team input:**

- Have you seen a physiotherapist/OT/SLT recently / ever?
- How have you found that (each) service?
- Do they come to the clinic you attend or do you have to go to them?
- Who referred you?

**An ‘excellent’ service:**

- Could you tell me what your ideal PD clinic would be like?
- What the most/least important things about PD services, to you?
- Are there any other services that you’ve heard about that you think might help you with your PD?
- Have you asked anyone about these? Who? Are they available to you?
- If you had to choose between a “bells and whistles” service that you had to travel for, and an ‘ok’ one near you, which would you choose?
- If you had to choose between a thorough clinic visit less often and a quick review more often, which would you choose?
- What do you dislike the most about the health services you have used?
- What if you got some reviews by telephone, maybe a bit more often but say, every second one was done by telephone?
- Would you consider using technology to aid your assessment/review?

**Family member (for carers specifically):**

- How are you finding the service for you? Is there a focus on your needs as the carer?

**Ending:**

Thank you for taking part. Before we wrap up:

- Is there anything else you would like to add about PD services and how they do, or don’t meet your needs?
- Is there anything that you feel is important about PD services in Ireland that we haven’t talked about?
